# Direct One-Step Growth of Bimetallic Ni_2_Mo_3_N on Ni Foam as an Efficient Oxygen Evolution Electrocatalyst

**DOI:** 10.3390/ma14164768

**Published:** 2021-08-23

**Authors:** Sang Heon Park, Soon Hyung Kang, Duck Hyun Youn

**Affiliations:** 1 Department of Chemical Engineering, Interdisciplinary Program in Advanced Functional Materials and Devices Development, Kangwon National University, Chuncheon 24341, Korea; parksh31@kangwon.ac.kr; 2 Department of Chemistry Education, Chonnam National University, Gwangju 61186, Korea

**Keywords:** water splitting, oxygen evolution reaction, bimetallic nitride, Ni_2_Mo_3_N, Ni foam

## Abstract

A simple and economical synthetic route for direct one-step growth of bimetallic Ni_2_Mo_3_N nanoparticles on Ni foam substrate (Ni_2_Mo_3_N/NF) and its catalytic performance during an oxygen evolution reaction (OER) are reported. The Ni_2_Mo_3_N/NF catalyst was obtained by annealing a mixture of a Mo precursor, Ni foam, and urea at 600 °C under N_2_ flow using one-pot synthesis. Moreover, the Ni_2_Mo_3_N/NF exhibited high OER activity with low overpotential values (336.38 mV at 50 mA cm^−2^ and 392.49 mV at 100 mA cm^−2^) and good stability for 5 h in Fe-purified alkaline electrolyte. The Ni_2_Mo_3_N nanoparticle surfaces converted into amorphous surface oxide species during the OER, which might be attributed to the OER activity.

## 1. Introduction

Hydrogen (H_2_) is a promising energy carrier due to its high mass-specific energy density (142 MJ kg^−1^), high utilization efficiency, and zero carbon emission when generated from renewable energy sources. Electrochemical water splitting from renewables such as solar or wind energy is considered a clean and efficient route for hydrogen production [1,2,3,4]. Water splitting consists of hydrogen evolution reaction (HER) and oxygen evolution reaction (OER). The four electrons involved in OER (4OH^−^ → 2H_2_O + O_2_ + 4e^−^) are kinetically sluggish relative to the two electrons involved in HER, requiring large overpotential values [5,6,7,8,9]. Ir- and Ru-based materials are typical catalysts for OER, but their high cost and scarcity restrict their widespread application [10,11,12]. Thus, developing alternative OER electrocatalysts based on low-cost and abundant materials is urgent for the large-scale proliferation of water-splitting systems.

Materials that include transition metals, such as transition metal oxides, transition metal nitrides (TMNs), and transition metal oxynitrides, demonstrated very promising OER activity [13,14]. Among them, various monometallic TMNs including Ni_3_N, Co_4_N, HfN, and Mn_3_N_2_ have been investigated as low-cost electrocatalysts [15,16,17,18]. TMNs possessing physical hardness, chemical stability, electrical conductivity, and unique electronic structure have been traditionally used as catalysts for chemical processes [19,20], and recently showed potential for energy applications [21,22]. However, these monometallic TMNs still exhibit limited OER performance. Designing bimetallic TMNs has proven to be an effective way to improve the OER performance of monometallic TMNs, which is expected to show the synergy between two distinct metal species [23,24]. In bimetallic TMNs, the presence of a second metal atom supplied more active sites and enhanced electronic conductivity, achieving higher OER activity compared to monometallic catalysts [25,26]. Among the various bimetallic TMNs, Ni-Mo nitrides have been extensively explored as OER electrocatalysts due to their high activity and stability. Although progress has been made, Ni-Mo nitrides are traditionally prepared by a complex method involving a two- or multistep annealing process. This typically involves hydrothermal Ni-Mo oxide formation and subsequent nitridation using NH_3_ gas for Ni-Mo nitride formation, making synthesis a challenge [27,28,29,30,31].

In this work, we report a simple and economical synthetic route for direct one-step growth of bimetallic Ni_2_Mo_3_N nanoparticles on Ni foam substrate (Ni_2_Mo_3_N/NF) for use as an OER catalyst. The Ni_2_Mo_3_N/NF catalyst was prepared by annealing Mo precursor, Ni foam, and urea at 600 °C under N_2_ flow in one pot. During annealing, inert N_2_ gas was used in exchange for toxic ammonia gas. In addition, no Ni precursor was added because Ni foam acted as the Ni source. Therefore, the suggested fabrication method is simple, economical, and eco-friendly. The resultant Ni_2_Mo_3_N/NF catalyst exhibited impressive OER catalytic performance with small overpotential values of 336.38 and 392.49 mV at current densities of 50 and 100 mA cm^−2^, respectively, and excellent stability over 5 h of operation at 50 mA cm^−2^. The high activity and stability with this simple synthetic method suggest that our Ni_2_Mo_3_N/NF catalyst could be a promising electrocatalyst for OER.

## 2. Materials and Methods

### 2.1. Materials

Molybdenum chloride (MoCl_5_) was purchased from Alfa Aesar. Urea (CH_4_N_2_O), ethanol (C_2_H_5_OH), and a 1.0 M potassium hydroxide (KOH) solution were purchased from Samchun. Notably, an Fe-free 1.0 M KOH electrolyte was prepared by following a previously reported method to avoid incidental Fe incorporation and consequent OER activity enhancement during the electrochemical tests [32]. For preparation of an Fe-free 1.0 M KOH solution, Ni(NO_3_)_2_·6H_2_O was dissolved in ultrapure water and 1.0 M KOH was added to precipitate high-purity Ni(OH)_2_. After three centrifugation and washing cycles, the high-purity Ni(OH)_2_ solid was mechanically stirred in 1.0 M KOH for at least 10 min and rested for 3 h. The mixture was centrifuged, and the purified KOH supernatant was transferred to a clean bottle and used as an Fe-free electrolyte. The Ni foam was purchased from Goodfellow (Ni003852), having a pore size of ca. 450 μm and a strut diameter of ca. 70 μm. Commercial IrO_2_ catalysts were purchased from Alfa Aesar (A17849).

### 2.2. Synthesis of Ni_2_Mo_3_N/NF

A measure of 3.66 mmol MoCl_5_ was dissolved in 2.53 mL ethanol, then 5.49 mmol urea (molar ratio of urea/Mo = 1.5) was added to the solution, which was stirred for 1 h until the urea was completely dissolved. The solution was transferred to an alumina boat with pieces of Ni foam and annealed at 600 °C (ramping at 3.3 °C min^−1^) for 3 h under flowing N_2_ gas (100 sccm) to fabricate the Ni_2_Mo_3_N/NF electrocatalyst.

### 2.3. Characterizations

A scanning electron microscope ((SEM, JEOL JSM-7900F) (Jeol, Peabody, MA, USA)) with an energy dispersive X-ray spectrometer (EDS) and a high-resolution transmission electron microscope ((HRTEM, JEM-2100F, JEOL (Acc. Voltage: 200 kV) (Jeol, Peabody, MA, USA)) were used to reveal detailed structural information. Crystalline structures of the prepared catalysts were investigated by X-ray diffraction (XRD, Miniflex 600, Rigaku, Tokyo, Japan) using Cu-Kα (wavelength = 1.5406 Å) radiation at 40 kV and 15 mA. Surface chemical states were analyzed using X-ray photoelectron spectroscopy (XPS, Thermo Fisher Scientific, K-Alpha, Waltham, MA, USA) with an X-ray source of Al-K_α1_. The recorded binding energies were calibrated using the adventitious carbon C 1s peak at 284.8 eV. In addition, the XPS spectra for Ni 2p and Mo 3d were deconvoluted to have area ratios of 1:2 (2p_1/2_:2p_3/2_) and 2:3 (3d_3/2_:3d_5/2_), respectively.

### 2.4. Electrochemical Tests

Electrochemical characterizations were carried out in a three-electrode cell system using a potentiostat ((PAR, VersaSTAT 4) (Ametek, Berwyn, PA, USA)) under an O_2_-purged Fe-free 1.0 M KOH solution. The Ni_2_Mo_3_N/NF (1 × 1 cm^2^) was directly used as a working electrode. The Ag/AgCl (3 M NaCl) and Pt wire were used as a reference and counter electrode, respectively. All potentials were converted to the reversible hydrogen electrode (RHE) using the equation (E_RHE_ = E_Ag/AgCl_ + 0.059 pH + E°_Ag/AgCl_). Linear sweep voltammetry (LSV) polarization curves were obtained using iR compensation at a scan rate of 5 mV s^−1^. The long-term stability test was carried out using the chronopotentiometric method. Electrochemical impedance spectroscopy (EIS) was performed from 10^5^ to 10^−1^ Hz with a modulation amplitude of 20 mV at 400 mV overpotential, and EIS plots were fitted with Z-view software.

## 3. Results and Discussion

Figure S1 shows digital photographs for the synthetic procedure. MoCl_5_ was dissolved in ethanol to form a dark-greenish solution (Figure S1a). At this step, MoCl_5_ reacts with ethanol vigorously, generating molybdenum orthoester and releasing HCl gas [33,34]. The addition of urea to the solution yielded a viscous Mo-urea complex (Figure S1b) [35,36]. Ni foams and Mo-urea complex were transferred to an alumina boat and annealed at 600 °C for 3 h under N_2_ flow (Figure S1c,d). Consequently, Ni_2_Mo_3_N nanoparticles grown directly on nickel foam were fabricated (Figure S1e,f). During the synthetic procedure, nitrogen was supplied from urea; thus, toxic ammonia gas was not employed for nitridation. Even more rewarding was that no Ni precursor was added for Ni_2_Mo_3_N generation because the Ni foam support acted as a Ni source through thermal diffusion [37,38,39,40]. These alterations in the technique enabled our synthetic method to be economical and straightforward.

Before further experiments, the annealing temperatures of Ni_2_Mo_3_N/NF samples were optimized. Figure S2 displays the XRD patterns of prepared samples at various annealing temperatures from 550 to 650 °C. A pure crystalline Ni_2_Mo_3_N phase was obtained only at an annealing temperature of 600 °C. At 650 °C, mixed phases of Ni_2_Mo_3_N and Mo_2_N were detected in XRD patterns, while at 550 °C, an oxide phase was observed with low peak intensities of Ni_2_Mo_3_N. Thus, the annealing temperature of 600 °C was employed as the optimum temperature condition to form Ni_2_Mo_3_N/NF.

**Figure 1:**
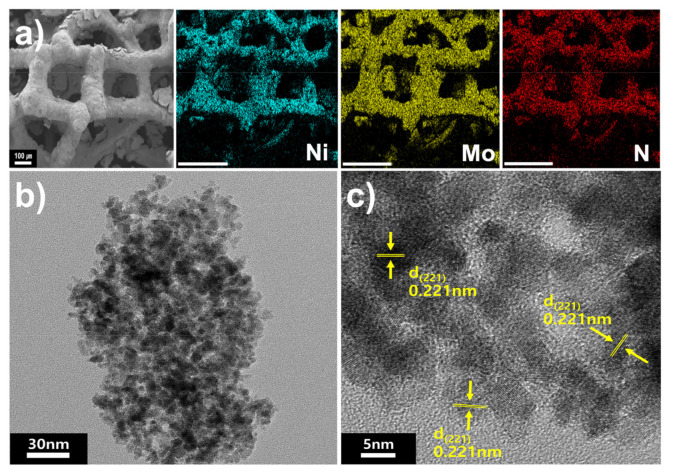
(**a**) SEM image of Ni_2_Mo_3_N/NF and SEM-EDS elemental mapping images (scale bar = 300 μm). (**b**,**c**) TEM images of Ni_2_Mo_3_N/NF.

Figure 1a shows SEM images of Ni_2_Mo_3_N/NF. A three-dimensional porous structure stems from the pristine Ni foam, and a rough surface originates from the growth of the Ni_2_Mo_3_N particles on the Ni foam. The elemental mapping image of Ni is consistent with Mo and N, indicating that the Ni_2_Mo_3_N nanoparticles are uniformly dispersed on the Ni foam substrate. Figure 1b and Figure S3 show the TEM image and the corresponding particle size distribution graph of Ni_2_Mo_3_N/NF, where 7.2 ± 1.1 nm Ni_2_Mo_3_N nanoparticles are observed without heavy aggregation. The observed lattice fringe of 2.21 Å in the high-resolution TEM (HRTEM) image (Figure 1c) corresponds to the Ni_2_Mo_3_N (221) plane.

**Figure 2:**
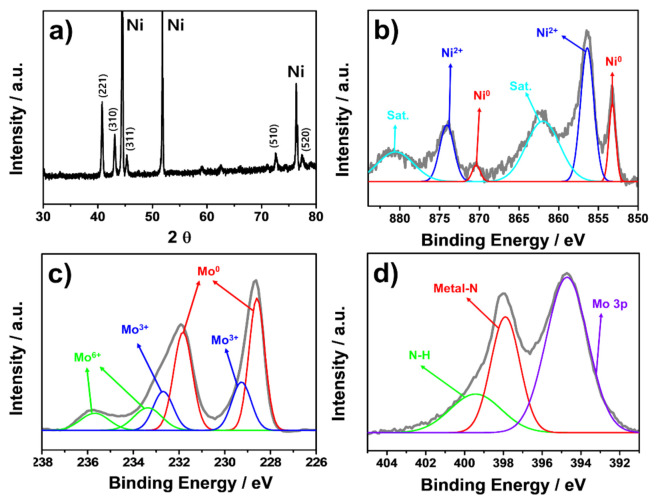
(**a**) XRD patterns of Ni_2_Mo_3_N/NF. XPS spectra of Ni_2_Mo_3_N/NF for (**b**) Ni 2p, (**c**) Mo 3d, and (**d**) N 1 s.

Figure 2a shows X-ray diffraction (XRD) patterns of Ni_2_Mo_3_N/NF. The intense peaks at 45, 52, and 76° can be indexed to metallic Ni (JCPDS no. 00-004-0850) from Ni foam. The other diffraction peaks observed at 40.7, 43.1, 45.3, 72.6, and 77.4° correspond to (221), (310), (311), (510), and (520) planes of reference in cubic Ni_2_Mo_3_N patterns (JCPDS no. 01-089-4564). No other phases such as MoO_3_ or Mo_2_N were detected; hence, phase-pure Ni_2_Mo_3_N was grown on the Ni foam. The Ni_2_Mo_3_N possesses a filled β-manganese structure composed of corner-sharing Mo_6_N octahedra and interpenetrated net-like Ni atoms [41,42].

The chemical states of Ni_2_Mo_3_N/NF were analyzed by X-ray photoelectron spectroscopy (XPS). Figure 2b shows the Ni 2p XPS spectra of Ni_2_Mo_3_N/NF. The peaks shown at 852.9 and 870.4 eV are ascribed to 2p_3/2_ and 2p_1/2_ of metallic Ni (Ni^0^), while the peaks centered at 856.4 and 873.9 eV are due to Ni^2+^ 2p_3/2_ and 2p_1/2_, respectively [27,33,43,44]. The high-resolution Mo 3d XPS spectra (Figure 2c) can be deconvoluted into three pairs with binding energies of 228.5/231.8, 229.4/232.6, and 233.4/235.7 eV corresponding to Mo^0^, Mo^3+^, and Mo^6+^, respectively [33,43,45]. The Mo^0^ and Mo^3+^ valence states originated from Ni_2_Mo_3_N, and the presence of Mo^6+^ is due to surface oxide formation [44,45,46,47]. In the N 1s XPS spectra (Figure 2d), the two deconvoluted peaks at 397.9 and 399.5 eV are attributed to metal-N and N-H groups, respectively. The N-H groups are likely associated with surface-adsorbed NH_x_ species due to reaction with moisture from air exposure [48]. In addition, the peak at 394.7 eV originated from partially overlapped Mo 3p [27,30,31].

**Figure 3:**
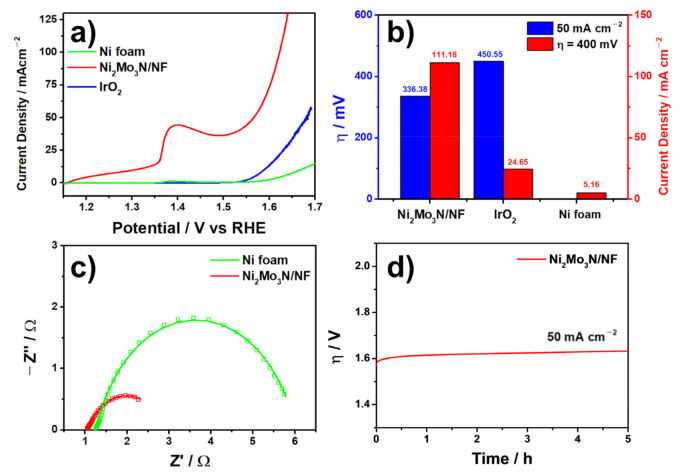
Electrochemical characterization of the prepared catalysts. (**a**) Polarization curves (1.0 M KOH solution), (**b**) bar graphs showing overpotentials at 50 mA cm^−2^ and current densities at an overpotential of 400 mV, (**c**) Nyquist plots, and (**d**) durability measurement.

Figure 3a exhibits the polarization curves for the OER with Ni_2_Mo_3_N/NF in an Fe-free 1.0 M KOH solution along with commercial IrO_2_ and pure Ni foam for comparison. The observed peak around 1.4 V for Ni_2_Mo_3_N/NF is ascribed to the oxidation of Ni(II)/Ni(III or IV) [49,50]. The Ni_2_Mo_3_N/NF exhibited a much higher current density over the whole potential region than the others. The overpotential values of Ni_2_Mo_3_N/NF at 50 mA cm^−2^ and 100 mA cm^−2^ were 336.38 mV (η_50_) and 392.49 mV (η_100_), respectively. The η_50_ value of Ni_2_Mo_3_N/NF was even smaller than 450.55 mV for commercial IrO_2_. The pure Ni foam did not reach 50 mA cm^−2^ in the measured potential range (Figure 3b) and showed poor OER activity with an η_10_ value of 358.91 mV, suggesting that the loaded Ni_2_Mo_3_N phase was mainly responsible for the OER activity. In addition, at an overpotential value of 400 mV, the current density of the Ni_2_Mo_3_N/NF reached 111.18 mA cm^−2^, which is 4.5 and 21.6 times higher than commercial IrO_2_ and Ni foam, respectively (Figure 3b). The Ni_2_Mo_3_N/NF recorded one of the best OER catalytic performances among reported TMN-based electrocatalysts (Table S1).

The electrochemical active surface area (ECSA), the area of the electrode materials that is accessible to the electrolyte for electrochemical reaction, was estimated by the double layer capacitance (C_dl_) method (Figure S4). The measured C_dl_ value for Ni_2_Mo_3_N/NF is 347.24 mF cm^−2^, whereas pure Ni foam and commercial IrO_2_ recorded small C_dl_ values of 0.68 and 0.55 mF cm^−2^, respectively. The high ECSA of Ni_2_Mo_3_N/NF suggests that the enhanced contact area between the catalyst and electrolyte is fruitful for improving the electrochemical activity of Ni_2_Mo_3_N/NF.

Electrochemical impedance spectroscopy (EIS) was conducted to characterize the prepared catalysts further, and the resulting Nyquist plots are presented in Figure 3c. A semicircle in the Nyquist plot represents the charge transfer resistance (R_ct_) and corresponding capacitance, describing the charge-transfer process at the catalyst/electrolyte interface. Generally, the R_ct_ value is inversely proportional to electrochemical activity. The Ni_2_Mo_3_N/NF catalyst exhibited a smaller R_ct_ value (1.861 Ω) than pure Ni foam (4.742 Ω), indicating enhanced OER catalytic activity due to the synergy between the Ni_2_Mo_3_N phase with high activity and the Ni foam providing a large surface area and high conductivity.

Chronopotentiometry tests were carried out to characterize the long-term stability of the OER, as it is an essential parameter for electrocatalysts. At 50 mA cm^−2^, the activity of Ni_2_Mo_3_N/NF was generally maintained for 5 h with a marginal overpotential increase, shown in Figure 3d. Therefore, the Ni_2_Mo_3_N/NF catalyst has excellent electrochemical activity and durability for the OER.

**Figure 4:**
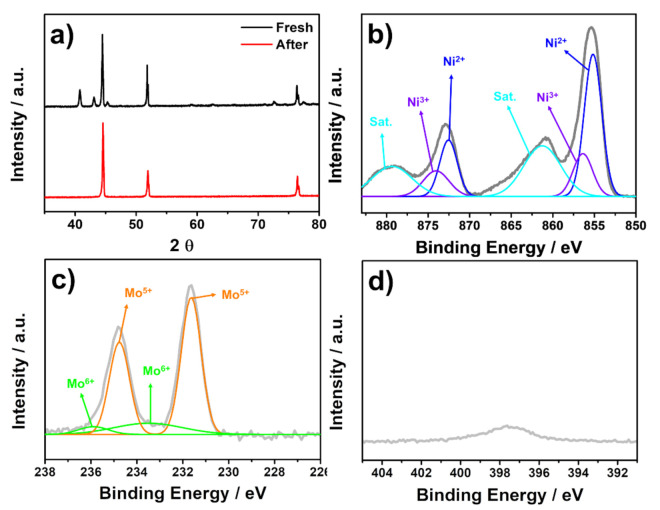
(**a**) XRD patterns of Ni_2_Mo_3_N/NF (fresh and after the durability test). XPS spectra of Ni_2_Mo_3_N/NF after the durability test. (**b**) Ni 2p, (**c**) Mo 3d, and (**d**) N 1s.

Further characterizations, including XRD, XPS, SEM, and TEM measurements, were conducted to monitor the structural changes of Ni_2_Mo_3_N/NF after the durability test. Figure 4a shows the XRD patterns of the Ni_2_Mo_3_N/NF catalyst after the 5 h durability test. The Ni_2_Mo_3_N peaks disappeared, and only metallic Ni peaks were observed, suggesting the transformation of crystalline Ni_2_Mo_3_N into an amorphous phase during the durability test. In the Ni 2p spectra of Ni_2_Mo_3_N/NF (Figure 4b), Ni^0^ peaks disappeared, and Ni^2+^ and Ni^3+^ peaks intensified, possibly due to the formation of NiO_x_ or NiOOH species. The peaks can be deconvoluted into three pairs with binding energies of 855.2/872.5, 856.4/874.2, and 861.3/879.4 eV corresponding to the 2p_3/2_/2p_1/2_ doublets of Ni^2+^, Ni^3+^, and satellites [27,44,45,51]. In Ni-containing catalysts, surface NiO_x_ species are generated in a low potential range below 1.35 V, which are further oxidized to NiOOH at ca. 1.4 V [52,53]. These NiO_x_ and NiOOH phases are indicated as a significant contributor to the OER performance [27,54]. The Mo 3d XPS spectra in Figure 4c show two pairs with binding energies of 231.7/234.9 and 233.5/235.9 eV, originating from Mo^5+^ and Mo^6+^ [43,55,56]. Additionally, the intensity of the N 1s spectra was significantly decreased after the durability test (Figure 4d). These results indicate the formation of amorphous surface oxide species from crystalline nitride species. However, in the Ar-sputtered Mo 3d and N 1s spectra of Ni_2_Mo_3_N/NF after the durability test (Figure S5), the nitride-related peaks appeared again [44,51]. In Figure S1a, Mo^0^ and Mo^3+^ peaks showed up as in the fresh Ni_2_Mo_3_N/NF sample, and the Mo 3d spectra showed four Mo oxidation states: Mo^0^ (228.3/231.9 eV), Mo^3+^ (229.5/232.6 eV), Mo^5+^ (232.0/235.0), and Mo^6+^ (233.5/235.8 eV) [43,44,55,56]. In Figure S1b, the intensity of the N 1s spectra increased where metal-N, N-H, and Mo 3p peaks were observed at 397.8, 399.5, and 394.8 eV, respectively [28,31]. These results lead us to conclude that the amorphous surface oxide species were formed after the OER tests, and the metal nitride species remained in the bulk.

**Figure 5:**
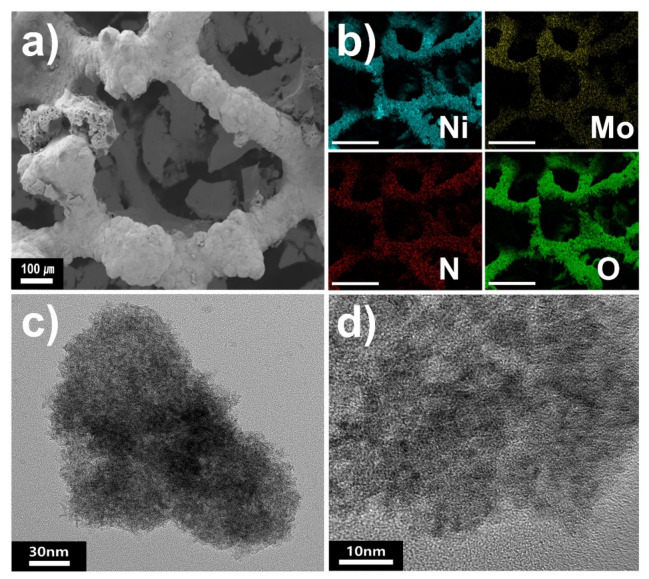
Ni_2_Mo_3_N/NF characterization results after the durability tests: (**a**) SEM images; (**b**) SEM-EDS elemental mapping images (scale bar = 300 μm); (**c**,**d**) TEM images.

SEM and TEM measurements were conducted to inspect the morphologies of the Ni_2_Mo_3_N/NF after the durability tests (Figure 5). The SEM image in Figure 5a shows that Ni_2_Mo_3_N remains on the Ni foam similar to fresh Ni_2_Mo_3_N/NF. SEM-EDS elemental mapping images indicate that the Ni, Mo, and N elements are still uniformly distributed over the Ni foam with a clear O presence due to the formation of amorphous surface oxide species. Nanoparticles less than 10 nm can be observed in the TEM images (Figure 5c,d) without noticeable aggregation. Additionally, the lattice structure of the particles was not observed, indicating the conversion of crystalline Ni_2_Mo_3_N/NF into amorphous phases.

## 4. Conclusions

We prepared Ni_2_Mo_3_N nanoparticles directly grown on Ni foam using one step, by annealing Ni foam, MoCl_5_, and urea in one pot. The resultant Ni_2_Mo_3_N/NF shows impressive electrocatalytic performance for OER in an Fe-purified alkaline electrolyte, with small overpotential values of 336.38 (η_50_) and 392.49 mV (η_100_) and good durability for 5 h. The OER tests revealed that the surface of Ni_2_Mo_3_N converted to amorphous surface oxide species, which might be responsible for its exceptional catalytic activity. Our work offers a facile and economical route for bimetallic nitrides and provides a new avenue for designing highly efficient electrocatalysts.

## Data Availability

The data presented in this study are available upon request from the corresponding author.

## Supplementary Materials

The following are available online at https://www.mdpi.com/article/10.3390/ma14164768/s1, Figure S1: Digital photographs for the synthetic procedure. Figure S2: XRD patterns of prepared samples with various annealing temperatures. Figure S3: Histogram showing the particle size distribution of Ni_2_Mo_3_N nanoparticles from the TEM images. Figure S4: Cyclic voltammograms of (a) Ni_2_Mo_3_N/NF, (b) pristine Ni foam and (c) IrO_2_ at different scan rates in 1.0 M KOH solution. (d–f) The corresponding current density versus scan rate plots showing C_dl_ values for Ni_2_Mo_3_N/NF, pristine Ni foam and IrO_2_. Figure S5: XPS spectra of Ni_2_Mo_3_N/NF after Ar-sputtering in the (a) Mo 3d and (b) N 1s, respectively. Table S1: Comparison of OER performances in alkaline media with reported TMN-based catalysts.
