# Supplementary material for: Direct One-Step Growth of Bimetallic Ni2Mo3N on Ni Foam as an Efficient Oxygen Evolution Electrocatalyst
Source: Materials (Basel). 2021 Aug 23;14(16):4768. doi: 10.3390/ma14164768 (PMC8398334; doi:10.3390/ma14164768)
Supplement: Supplementary file 1 [file materials-14-04768-s001.zip › materials-1296248-supplementary.pdf]

materials

MDPI

Supplementary Materials

Direct One-Step Growth of Bimetallic Ni_2Mo_3N on Ni Foam as an Efficient Oxygen Evolution Electrocatalyst

Sang Heon Park [1], Soon Hyung Kang [2,*] and Duck Hyun Youn [1,*]

[1] Department of Chemical Engineering, Interdisciplinary Program in Advanced Functional Materials and Devices Development, Kangwon National University, Chuncheon 24341, Korea; parksh31@kangwon.ac.kr
[2] Department of Chemistry Education, Chonnam National University, Gwangju 61186, Korea
* Correspondence: skang@jnu.ac.kr (S.H.K.); youndh@kangwon.ac.kr (D.H.Y.)

Citation: Park, S.H.; Kang, S.H.; Youn, D.H. Direct One-Step Growth of Bimetallic Ni_2Mo_3N on Ni Foam as an Efficient Oxygen Evolution Electrocatalyst. *Materials* **2021**, *14*, 4768. https://doi.org/10.3390/ma14164768

Academic Editor: Ivano E. Castelli

Received: 28 June 2021
Accepted: 19 August 2021
Published: 23 August 2021

Publisher's Note: MDPI stays neutral with regard to jurisdictional claims in published maps and institutional affiliations.

Copyright: © 2021 by the authors. Licensee MDPI, Basel, Switzerland. This article is an open access article distributed under the terms and conditions of the Creative Commons Attribution (CC BY) license (http://creativecommons.org/licenses/by/4.0/).

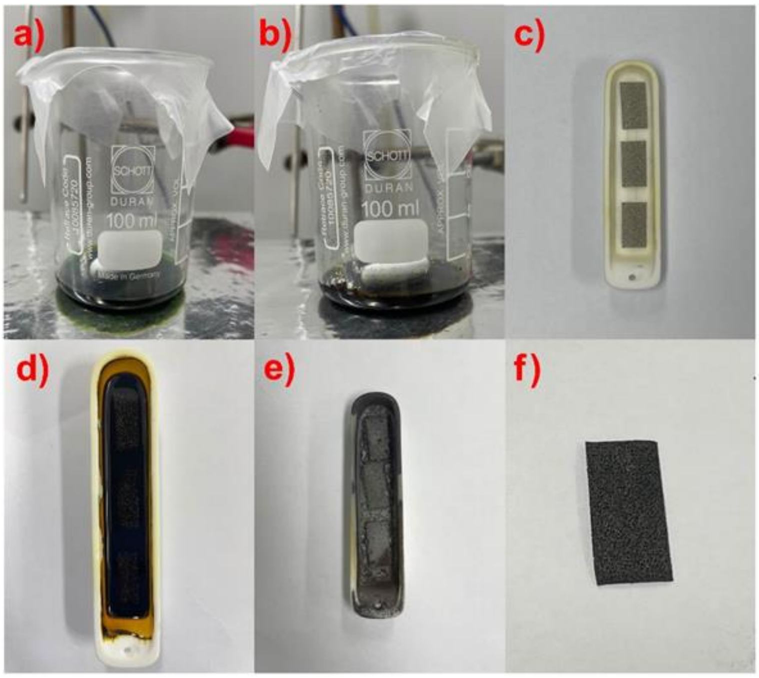

Figure S1. Digital photographs for the synthetic procedure: (**a**) $MoCl_5$ was dissolved in ethanol to form a dark-greenish solution; (**b**) The addition of urea to the solution yielded a viscous Mo-urea complex; (**c,d**) Ni foams and Mo-urea complex were transferred to an alumina boat and annealed at 600 °C for 3 h under N_2 flow; (**e,f**) Consequently, Ni_2Mo_3N nanoparticles grown directly on nickel foam were fabricated.

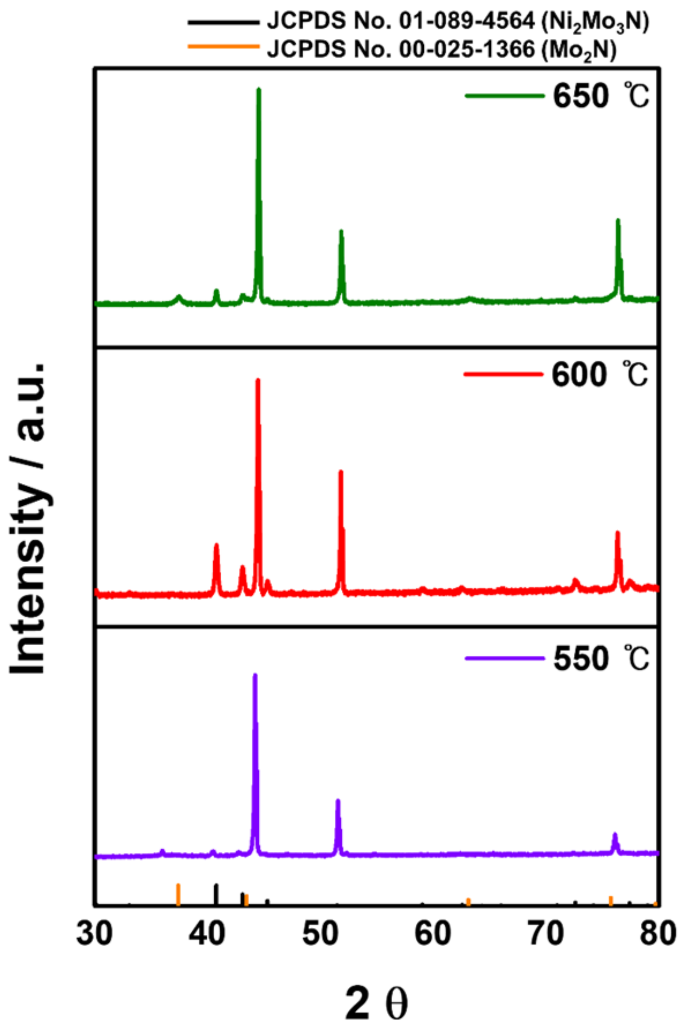

Figure S2. XRD patterns of prepared samples with various annealing temperatures.

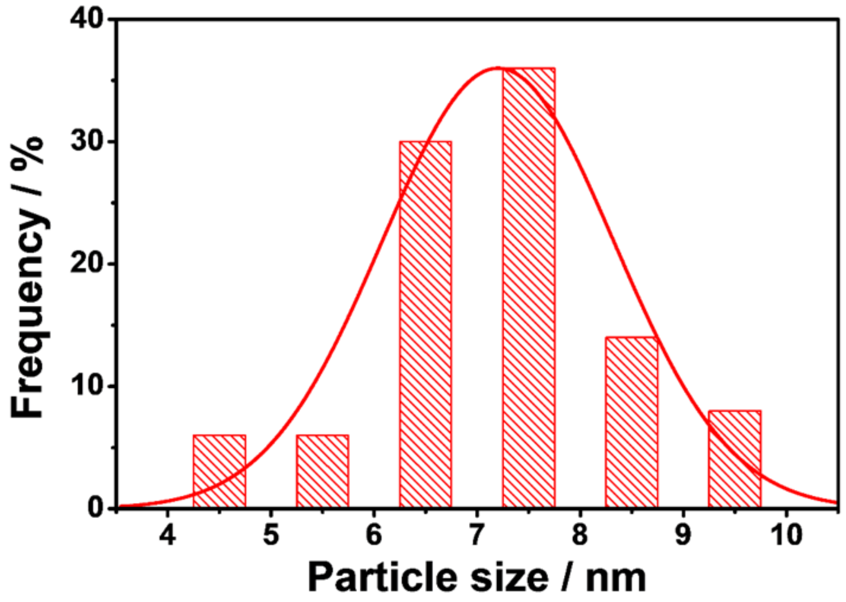

Figure S3. Histogram showing the particle size distribution of Ni_2Mo_3N nanoparticles from the TEM images.

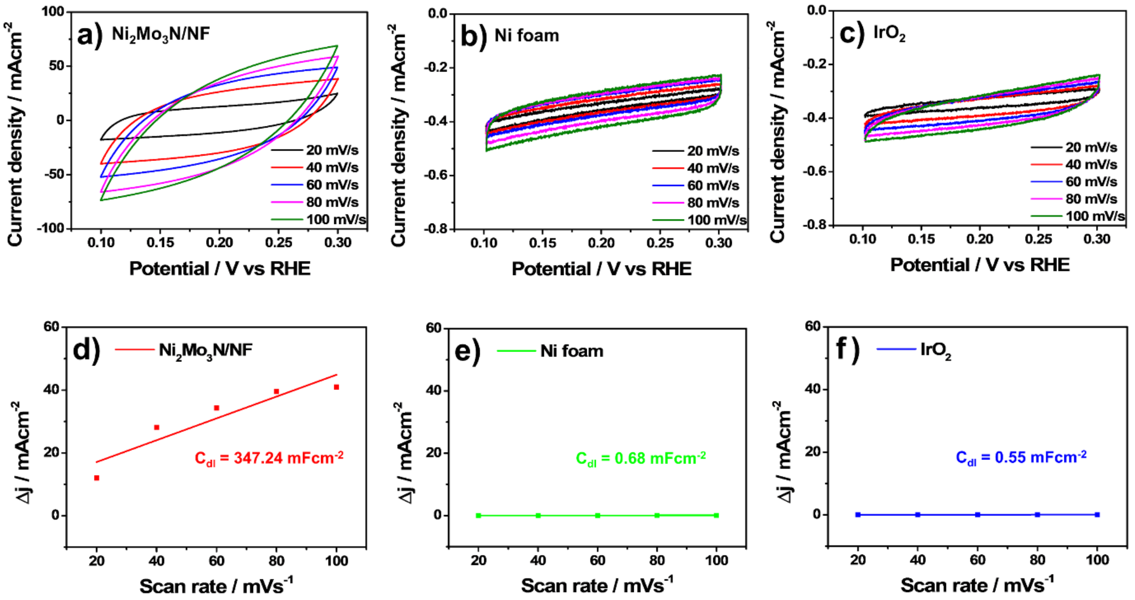

Figure S4. Cyclic voltammograms of a) Ni_2Mo_3N/NF, b) pristine Ni foam and c) IrO_2 at different scan rates in 1.0 M KOH solution. d-f) The corresponding current density versus scan rate plots showing C_{dl} values for Ni_2Mo_3N/NF, pristine Ni foam and IrO_2.

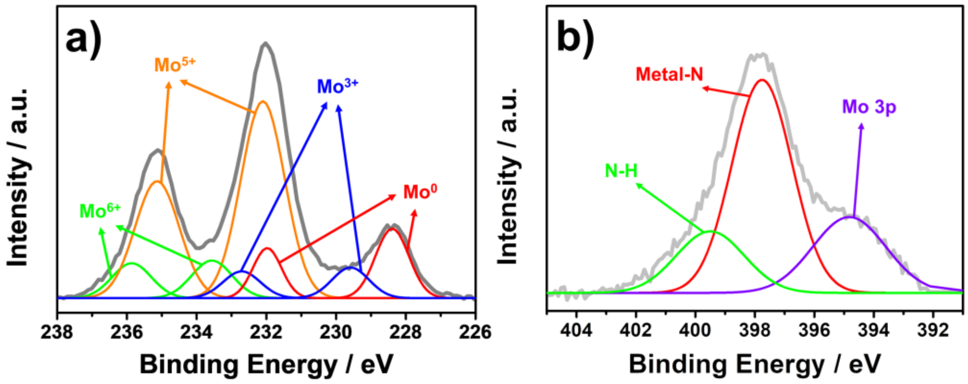

Figure S5. XPS spectra of Ni_2Mo_3N/NF after Ar-sputtering in the a) Mo 3d and b) N 1s, respectively.

Table S1. Comparison of OER performances in alkaline media with reported TMN-based catalysts.

Catalyst	η_{10} (mV)	η_{50} (mV)	η_{100} (mV)	Electrolyte	Ref.
Ni_2Mo_3N/NF		336.38	392.49	1 M KOH	This work
Co-Mo_2N	302	400	≒ 485	1 M KOH	[1]
Ni/$Ni_{0.2}Mo_{0.8}N$@N-C	260	≒ 370		1 M KOH	[2]
Zn-Ni_3FeN/NG	370			0.1 M KOH	[3]
NiMoN-550	295	≒ 365	≒ 390	1 M KOH	[4]
CuFeN/CNT		≒ 375	392	1 M KOH	[5]
Ni_3FeN-NPs	241	≒ 340	≒ 430	1 M KOH	[6]
NiCoN/CC	360	≒ 400		1 M NaOH	[7]
VN-Co-P	335	≒ 460		1 M KOH	[8]
Ni_3FeN	355	≒ 560		0.1 M KOH	[9]
NiCo-nitrides/$NiCo_2O_4$/GF	183	344	≒ 430	1 M KOH	[10]
PF/$Ni_{1.5}Co_{1.5}N$	280	≒ 380		1 M KOH	[11]
Co_4N	330	≒ 375		1 M KOH	[12]

References.

1. Shi, X.; Wu, A.; Yan, H.; Zhang, L.; Tian, C.; Wang, L.; Fu, H. A "MOFs plus MOFs" strategy toward Co-Mo_2N tubes for efficient electrocatalytic overall water splitting. *J. Mater. Chem. A* **2018,** *6*, 20100-20109.
2. Li, T.; Hu, Y.; Pan, X.; Yin, J.; Li, Y.; Wang, Y.; Zhang, Y.; Sun, H.; Tang, Y. N-carbon supported hierarchical Ni/$Ni_{0.2}Mo_{0.8}N$ nanosheets as high-efficiency oxygen evolution electrocatalysts. *Chem. Eng. J.* **2020,** *392*, 124845.
3. He, X.; Tian, Y.; Huang, Z.; Xu, L.; Wu, J.; Qian, J.; Zhang, J.; Li, H. Engineering the electronic states of Ni_3FeN via zinc ion regulation for promoting oxygen electrocatalysis in rechargeable Zn-air batteries. *J. Mater. Chem. A* **2021,** *9*, 2301-2307.
4. Yin, Z.; Sun, Y.; Zhu, C.; Li, C.; Zhang, X.; Chen, Y. Bimetallic Ni-Mo nitride nanotubes as highly active and stable bifunctional electrocatalysts for full water splitting. *J.Mater. Chem. A* **2017,** *5*, 13648-13658.
5. Choi, Y.; Kim, D.; Lin, L.; Yan, B.; Hong, H.; Qin, X.; Piao, Y. CuFeN/CNT composite derived from kinetically modulated urchin-shaped MOF for highly efficient OER catalysis. *Electrochim. Acta* **2021,** *389*, 138637.
6. Chen, Q.; Wang, R.; Yu, M.; Zeng, Y.; Lu, F.; Kuang, X.; Lu, X. Bifunctional iron-nickel nitride nanoparticles as flexible and robust electrode for overall water splitting. *Electrochim. Acta* **2017,** *247*, 666-673.
7. Han, L.; Feng, K.; Chen, Z. Self-supported cobalt nickel nitride nanowires electrode for overall electrochemical water splitting. *Energy Technol.* **2017,** *5*, 1908-1911.
8. Yang, H.; Hu, Y.; Huang, D.; Xiong, T.; Li, M.; Balogun, M.-S.; Tong, Y. Efficient hydrogen and oxygen evolution electrocatalysis by cobalt and phosphorus dual-doped vanadium nitride nanowires. *Mater. Today Chem.* **2019,** *11*, 1-7.
9. Fu, G.; Cui, Z.; Chen, Y.; Xu, L.; Tang, Y.; Goodenough, J. B. Hierarchically mesoporous nickel-iron nitride as a cost-efficient and highly durable electrocatalyst for Zn-air battery. *Nano Energy* **2017,** *39*, 77-85.
10. Liu, Z.; Tan, H.; Liu, D.; Liu, X.; Xin, J.; Xie, J.; Zhao, M.; Song, L.; Dai, L.; Liu, H. Promotion of overall water splitting activity over a wide pH range by interfacial electrical effects of metallic NiCo-nitrides nanoparticle/$NiCo_2O_4$ nanoflake/graphite fibers. *Adv. Sci.* **2019,** *6*, 1801829.
11. Bai, X.; Wang, Q.; Xu, G.; Ning, Y.; Huang, K.; He, F.; Wu, Z. j.; Zhang, J. Phosphorus and fluorine Co-doping induced enhancement of oxygen evolution reaction in bimetallic nitride nanorods arrays: Ionic liquid-driven and mechanism clarification. *Chem. Eur. J.* **2017,** *23*, 16862-16870.
12. Chen, P.; Xu, K.; Tong, Y.; Li, X.; Tao, S.; Fang, Z.; Chu, W.; Wu, X.; Wu, C. Cobalt nitrides as a class of metallic electrocatalysts for the oxygen evolution reaction. *Inorg. Chem. Front.* **2016,** *3*, 236-242.
